# DeepCNF-D: Predicting Protein Order/Disorder Regions by Weighted Deep Convolutional Neural Fields

**DOI:** 10.3390/ijms160817315

**Published:** 2015-07-29

**Authors:** Sheng Wang, Shunyan Weng, Jianzhu Ma, Qingming Tang

**Affiliations:** 1 Department of Human Genetics, University of Chicago, Chicago, IL 60637, USA; 2 Toyota Technological Institute at Chicago, Chicago, IL 60637, USA; E-Mails: majianzhu@gmail.com (J.M.); tqm2004@gmail.com (Q.T.); 3 MoE Key Laboratory of Developmental Genetics and Neuropsychiatric Diseases, Bio-X Center, School of Life Sciences and Biotechnology, Shanghai Jiao Tong University, Shanghai 200240, China; E-Mail: wengshunyan@hotmail.com

**Keywords:** intrinsically disordered proteins, prediction of disordered regions, machine learning, deep learning, deep convolutional neural network, conditional neural field

## Abstract

Intrinsically disordered proteins or protein regions are involved in key biological processes including regulation of transcription, signal transduction, and alternative splicing. Accurately predicting order/disorder regions *ab initio* from the protein sequence is a prerequisite step for further analysis of functions and mechanisms for these disordered regions. This work presents a learning method, weighted DeepCNF (Deep Convolutional Neural Fields), to improve the accuracy of order/disorder prediction by exploiting the long-range sequential information and the interdependency between adjacent order/disorder labels and by assigning different weights for each label during training and prediction to solve the label imbalance issue. Evaluated by the CASP9 and CASP10 targets, our method obtains 0.855 and 0.898 AUC values, which are higher than the state-of-the-art single *ab initio* predictors.

## 1. Introduction

Protein sequence determining structure and therefore function is a long-standing paradigm in biology. However, this paradigm has been challenged ever since the discovery of the first functional protein featuring no stable structure over 50 years ago [1]. Since then, scientists have been discovering more and more proteins and/or protein regions with no unique structure that are involved in key biological processes, such as regulation of transcription, signal transduction, cell cycle control, post translational modifications, ligand binding, protein interaction, and alternative splicing [2,3]. Formally, these proteins and/or protein regions are defined as Intrinsically Disordered Proteins (IDP) or Intrinsically Disordered Regions (IDR).

Since the late 1990s, more interest has been focused on IDP and IDR due to additional evidence from experimental approaches such as NMR [4]. Due to the laborious and expensive nature of these experiments, numerous computational approaches have been proposed to predict the order/disorder regions [5]. Briefly, these methods could be categorized into two groups, (a) single *ab initio* methods that require only the protein sequence as input, and results are predicted based on a single method, such as IUpred [6], DISOPRED [7], SPINE-D [8], and DNdisorder [9]; (b) hybrid or consensus methods that exploit more information such as templates or output from other algorithms. Examples include PrDOS [10], PONDR [11], POODLE [12], and MetaDisorder [13]. For a more detailed introduction for these and other prediction methods, please refer to excellent reviews written by He *et al.* and Deng *et al.* [5,14].

Despite the good performance of currently available hybrid and consensus methods for order/disorder prediction [15], an accurate single *ab initio* method is still valuable since the improved results may contribute to other consensus methods, and then in turn to increase overall prediction accuracy. However, there are three challenges that block the advancement of single methods: (a) How to exploit the interdependency of adjacent order/disorder states (or labels), as well as the long-range sequential information that determines the order/disorder state at a certain position. In fact, adjacent order/disorder states are interdependent. In other words, the order/disorder state in one position influences adjacent states, as shown by the occurrence of long consecutively disordered regions. PrDOS-CNF [15] and OnD-CRF [16] exploit this interdependency between adjacent order/disorder labels through a linear-chain Conditional Random Field structure [17], but they are unable to describe the long-range sequence information; (b) How to solve the label imbalance issue. As the frequencies of order/disorder labels are strongly imbalanced (only ~6% of residues are disordered), using the conventional training method would bias the model towards fitting ordered states while performing poorly for disordered states; (c) How to incorporate additional features besides traditional ones. In short, it has been demonstrated that charged and hydrophilic residues, such as P, E, S, Q and K are more likely to be in disordered states, whereas neutral and hydrophobic residues, such as C, W, I, Y, F, L, M and H, are order-promoting residues [2]. It has also been suggested that evolutionary conservation largely determines order/disorder states [18]. Besides these amino acid and evolution related features, we need to find additional relevant and/or complementary features that could contribute to the order/disorder prediction accuracy.

This paper presents a machine learning model, called weighted DeepCNF (Deep Convolutional Neural Fields), for order/disorder region prediction to overcome the three challenges described above. (a) Weighted DeepCNF combines the advantages of both conditional neural fields (CNF) [19] and deep convolutional neural networks [20]. It not only captures medium- and long-range sequence information, but also describes interdependency of order/disorder labels among adjacent residues; (b) To solve the label imbalance issue, the reciprocal of the occurrence frequencies of order/disorder labels were used for weighting during training and prediction; (c) Besides the traditional amino acid and evolution related features that are relevant to order/disorder discrimination, a strong tendency has been shown for disordered regions to exist in the coiled state [18] and exposed to solvent [2]. Inspired by this phenomenon, additional structural properties, such as predicted secondary structure [21] and predicted solvent accessibility [22], were incorporated into our weighted DeepCNF model. Finally, experiments have shown that our method outperforms the state-of-the-art methods on publically available Critical Assessment of protein Structure Prediction (CASP) [15] dataset in terms of Area Under the ROC Curve (AUC) value.

## 2. Results

In this section we describe our experimental verification on the prediction for disordered regions. The first part describes the datasets we use, the programs to be compared with and the evaluation measures. The second part shows how we train and validate our DeepCNF model using a small Disorder723 dataset [23] by 10 cross validations to find out the best combination of the model parameters. In the third part, based on the best parameters determined from the Disorder723 dataset, we trained a model using a large but non-redundant UniProt90 dataset [24], and evaluated our method DeepCNF-D (here “D” stands for Disorder) with the other state-of-the-art programs on two publicly available CASP datasets [15,25].

### 2.1. Dataset, Programs to Be Compared, and Evaluation Measures

#### 2.1.1. Training, Validation, and Evaluation Dataset

Our weighted DeepCNF model for order/disorder prediction is trained and validated using Disorder723 dataset. This dataset, built by Cheng *et al.* [23] in May 2004, consists of 723 non-redundant chains which span at least 30 amino acids and were solved by X-ray diffraction with a resolution of around 2.5 Å.

A ten-fold cross-validation on this Disorder723 dataset was performed to determine the model parameter. In particular, the original dataset was randomly partitioned into 10 equal-sized subsamples. Among the 10 subsamples, a single subsample is retained as the validation data for testing the model, while the remaining nine are used as training data. The list of all these training/validating proteins, as well as the corresponding features for our model, could be found in Supplemental Data.

We further trained our weighted DeepCNF model using UniProt90 dataset for release purpose and evaluated it with two publicly available CASP datasets, *i.e.*, CASP9 [25] and CASP10 [15]. CASP9 dataset contains 117 sequences while CASP10 contains 94. The sequences from UniProt90 dataset were downloaded from MobiDB [24], followed up by a 25% sequence identity filter to those sequences from the two CASP datasets. The number of remaining sequences for training our model is 20,337. This UniProt90 training list, as well as the list and features for CASP datasets, can be found in Supplemental Data. Note that the determination of disordered residues in the UniProt90 dataset follows the same procedure as Cheng *et al.* [23] for Disorder723 dataset.

We also analyzed the overall properties of the non-terminal disordered regions with different lengths on the four datasets used in this work. As shown in Table 1, although the testing datasets (*i.e.*, CASP9 and CASP10) used in this study are dominated by proteins with short disordered regions, the two training datasets (*i.e*., Disorder723 and UniProt90) are relatively equally distributed in disordered region length, especially the UniProt90 dataset which is used to train our model for release.

**Table 1:** Overall properties of the non-terminal disordered regions with different lengths on the four datasets used in this work.

Datasets	Length of Disordered Regions				Number of Fragments of Disordered Regions			
	1–5	6–15	16–25	>25	1–5	6–15	16–25	>25
Disorder723	964	2083	883	852	492	226	45	19
UniProt90	12,804	37,420	16,646	22,655	4133	4093	852	514
CASP9	272	494	215	119	118	52	11	3
CASP10	163	261	113	55	73	31	6	2

#### 2.1.2. Programs for Comparison

We compared our method with the following programs: IUpred [6], SPINE-D [8], and DISOPRED3 [7]. IUpred has two different prediction modes: IUpred (long) and IUpred (short). The former is designed for long disordered regions while the latter for short ones, and both modes are only based on the amino acid related features; SPINE-D and DISOPRED3 rely not only on amino acid related features, but also on evolution related features generated by PSI-BLAST. SPINE-D relies further on structure related features generated by SPINE-X [26]. It should be noted that all these programs to be compared are *ab initio* methods and are downloadable. Since our method is also an *ab initio* method, it might be unfair to compare ours with those clustering, template-based, and meta or consensus methods as defined in Deng *et al.* [14]. All results from IUpred, SPINE-D, and DISOPRED3 on the corresponding datasets can be found in Supplemental Data.

#### 2.1.3. Evaluation Measurement

For evaluation of disorder predictors as binary classifiers we used the precision defined as TP/(TP + FP), the balanced accuracy (bacc) defined as 0.5×(TP/(TP+FN)+TN/(TN+FP)), and the Matthews correlation coefficient (MCC) defined as $\frac{\text{TP}\times \text{TN}-\text{FP }\times \text{ FN}}{\sqrt{\left(\text{TP }+\text{ FP}\right)\left(\text{TN }+\text{ FP}\right)\left(\text{TP }+\text{ FN}\right)\left(\text{TN }+\text{ FN}\right)}}$, where TP (True Positives) and TN (True Negatives) are the numbers of correctly predicted disordered and ordered residues, respectively, whereas FP (False Positives) and FN (False Negatives) are the numbers of misclassified disordered and ordered residues, respectively [15].

As the problem of order/disorder region prediction is strongly imbalanced (only ~7% of residues are disordered), using the conventional accuracy and precision measurement might inflate performance estimates and is therefore not appropriate [27]. Another paradoxical issue is the decision threshold for order/disorder classification. Depending on how users set the threshold, a bias might be introduced, which could lead to an unfair comparison between distinct studies. To solve this issue, we consider the Receiver Operating Characteristic (ROC) analysis for the assessment of protein disorder predictions [27]. An ROC curve represents a monotonic function describing the balance between the True Positive (TP) and False Positive (FP) rates of a predictor. Since all the programs in this study could output a probability value, for a set of probability thresholds (from 0 to 1), a residue is considered as a positive case (*i.e*., disordered) if its predicted probability is equal to or greater than the threshold value. The area under the curve (AUC) is used as an aggregate measure of the overall quality of a prediction method. An area of 1.0 corresponds to a perfect predictor while an area of 0.5 means purely random.

### 2.2. Determining the Best Model Parameters on Disorder723 Dataset

There are three components of tunable parameters in the weighted DeepCNF model, (a) number of hidden layers; (b) weight of labels; and (c) combination of input features. It should be noted that in order to simplify the following analysis, we fix some of the other tunable model parameters such as the window size (fixed to 11) and the number of neurons at each layer (fixed to 50). Thus, by default, our weighted DeepCNF model contains two hidden layers, applies 0.7:9.3 for the weight ratio between order and disorder labels, and includes all 129 features from the three classes.

#### 2.2.1. Contributions of Number of Layers

To show the relationship between the performance and the number of layers, we trained three different weighted DeepCNF models with one, two, and three layers, respectively. As shown in Table 2, the best AUC value is obtained by using two layers. The reason for getting inferior results when using three layers compared to using two layers may be due to over-fitting because the regularization factor (fixed at 200) is the same for all trained models. If the regularization factor is fine-tuned specifically for the three layer model, the results could be comparable to the two layer model. However, since the three layer model contains much more parameters than the two layer model, while achieving a similar result in terms of AUC value with much lower efficiency, we decided to use the two layer model in the following analysis.

**Table 2:** AUC values of different layer models on 10 cross validation batch datasets of Disorder723. Note that other model parameters are fixed as default. The best value is shown in bold (the same convention is used in Table 3, Table 4, Table 5, Table 6 and Table 7).

Number of Hidden Layers	AUC Value of 10 cross Validation Batch Datasets										
	1	2	3	4	5	6	7	8	9	10	Mean
1	0.878	0.925	0.842	0.853	0.882	0.835	0.871	0.868	0.898	0.842	0.869
2	**0.904**	**0.947**	**0.875**	**0.886**	**0.917**	**0.861**	**0.903**	**0.909**	**0.939**	**0.868**	**0.901**
3	0.887	0.936	0.863	0.873	0.902	0.852	0.887	0.908	0.923	0.857	0.889

#### 2.2.2. Contributions of Different Weight Ratios

It is well known that the label imbalance issue occurs in many real-world prediction tasks [27]. A simple solution to address this problem is to assign different weights to different labels [28]. Here we show that by assigning the reciprocal of the occurrence frequency as the weight for order/disorder label, the trained model reaches the best performance compared to an equally weighted label ratio. Specifically, as shown in Table 3, the 1:1 weight ratio obtains a mean AUC value of 0.884, while the reciprocal of the occurrence frequency of the order/disorder label (0.7:9.3 weight ratio) obtains 0.901. However, there are no significant differences between weight ratios 1:9, 0.5:9.5, and 0.7:9.3. These results may imply that by assigning the weight ratio around the closer range of the reciprocal ratio there will not be a large difference in the performance.

**Table 3:** AUC values of different combinations of weight ratio between order and disorder states on 10 cross validation batch datasets of Disorder723.

Weight Ratio	AUC Value of 10 cross Validation Batch Datasets										
	1	2	3	4	5	6	7	8	9	10	Mean
5:5	0.884	0.932	0.857	0.871	0.902	0.842	0.884	0.895	0.921	0.850	0.884
2:8	0.892	0.938	0.863	0.882	0.911	0.853	0.891	0.902	0.928	0.857	0.892
1:9	0.899	0.943	0.869	**0.887**	0.915	0.858	0.897	**0.911**	0.933	0.862	0.897
0.7:9.3	**0.904**	0.947	**0.875**	0.886	0.917	**0.861**	**0.903**	0.909	0.939	**0.868**	**0.901**
0.5:9.5	0.901	**0.948**	0.872	0.884	**0.919**	0.857	0.902	0.903	**0.942**	0.864	0.899

#### 2.2.3. Contributions of Different Combinations of Feature Classes

As mentioned in the previous section, the features used in the training process consist of three classes: evolution related, structure related, and amino acid related, respectively. In order to estimate the impact of each class on order/disorder prediction, we applied each one and several combinations of them to train the model and perform the prediction. Table 4 illustrates the AUC value of 10 cross validation batch datasets of Disorder723 with settings of different combinations of feature classes. Not surprisingly, the most contributing feature class is evolutionary information, which confirms that it is the conservation profile that makes the order/disorder regions different [18]. The second important feature class is the structural information class which is based on predicted secondary structure and solvent accessibility. It is interesting that, although structure related features are actually derived from evolutionary information, the combination of these two classes of features reaches 0.889 mean AUC. This phenomenon was reported in [9], with the possible interpretation that there is a strong tendency for disorder regions to exist in the coiled state [18] and to be exposed to solvent [2]. Finally, using amino acid related feature alone reaches 0.833 mean AUC. The combination of all three classes of features achieves the best AUC value for each cross validation batch dataset.

**Table 4:** Contribution of different combinations of feature classes for the AUC value on 10 cross validation batch datasets of Disorder723.

Feature Class	AUC Value of 10 cross Validation Batch Datasets										
	1	2	3	4	5	6	7	8	9	10	Mean
Amino acid	0.843	0.877	0.793	0.832	0.845	0.784	0.852	0.834	0.865	0.803	0.833
Structural	0.864	0.904	0.830	0.841	0.858	0.826	0.863	0.858	0.882	0.819	0.855
Evolution	0.874	0.920	0.836	0.857	0.879	0.832	0.876	0.880	0.908	0.834	0.870
Amino acid + Evolution	0.883	0.928	0.845	0.866	0.887	0.843	0.884	0.885	0.917	0.847	0.879
Structural + Evolution	0.895	0.935	0.868	0.873	0.901	0.850	0.897	0.896	0.924	0.859	0.889
All features	**0.904**	**0.947**	**0.875**	**0.886**	**0.917**	**0.861**	**0.903**	**0.909**	**0.939**	**0.868**	**0.901**

### 2.3. Comparison with Other Methods on Disorder723 and CASP Datasets

#### 2.3.1. Performance on Disorder723 Dataset

Table 5 shows the performance of our method DeepCNF-D and the other four methods in terms of AUC value on the 10 cross validation batch datasets of Disorder723. As listed in this table, for eight of the ten batches, our method outperforms all the other methods; for the remaining two batches, our method is comparable to SPINE-D and only inferior to DisoPred3. Note that DisoPred3 is the current state-of-the-art method and is trained on a much larger PDB90 dataset [29]. However, DeepCNF-D achieves the best performance in terms of the mean AUC value at 0.901, which is better than the state-of-the-art value achieved on this Disorder723 dataset by DNdisorder at AUC value 0.899 [9]. Just by using pure amino acid feature shown in Table 4, our DeepCNF model could reach the mean AUC value 0.833, which is higher than IUpred (0.810 and 0.721 for long and short prediction modes, respectively). Note that IUpred is the current best order/disorder predictor based on amino acid sequence only.

**Table 5:** AUC value of several methods on 10 cross validation batch datasets of Disorder723.

Methods	AUC Value of 10 cross Validation Batch Datasets										
	1	2	3	4	5	6	7	8	9	10	Mean
Iupred (long)	0.747	0.764	0.645	0.758	0.727	0.702	0.732	0.694	0.747	0.689	0.721
Iupred (short)	0.821	0.857	0.756	0.826	0.823	0.752	0.840	0.787	0.839	0.795	0.810
SPINE-D	0.885	0.929	0.885	0.888	0.897	0.848	0.877	0.906	0.914	0.838	0.887
DisoPred3	0.894	0.932	**0.892**	**0.920**	0.910	0.846	0.879	0.896	0.917	0.840	0.893
DeepCNF-D	**0.904**	**0.947**	0.875	0.886	**0.917**	**0.861**	**0.903**	**0.909**	**0.939**	**0.868**	**0.901**

#### 2.3.2. Performance on CASP Dataset

Table 6 and Table 7 summarize the performances of our method DeepCNF-D and the other four predictors on the publically available CASP9 and CASP10 datasets. In order to perform a fair comparison with DisoPred3 and SPINE-D, which are trained on PDB90 and PDB25 datasets respectively, we re-trained our weighted DeepCNF model using the UniProt90 dataset from MobiDB with removal of the overlap sequences with CASP datasets by a 25% sequence identity filter. It is observed that DeepCNF-D reaches 0.855 AUC value on CASP9 and 0.898 on CASP10, which are higher than DisoPred3, SPINE-D, and IUpred for both long and short prediction modes. Moreover, if only amino acid related features are used, DeepCNF-D (ami_only) achieves 0.7 AUC on CASP9 and 0.772 on CASP10, which are significantly higher than IUpred, though with extremely fast running speed.

**Table 6:** Performance of several predictors on CASP9. We show average value for balanced accuracy (bacc), precision, Mattehews correlation coefficient (MCC), and Area under the ROC curve (AUC).

Predictor	Precision	Bacc	MCC	AUC
Iupred (long)	0.238	0.546	0.118	0.567
Iupred (short)	0.433	0.698	0.342	0.657
SPINE-D	0.382	**0.769**	0.391	0.832
DisoPred3	**0.665**	0.704	0.464	0.842
DeepCNF-D	0.598	0.752	**0.486**	**0.855**
DeepCNF-D (ami_only)	0.549	0.707	0.400	0.700

**Table 7:** Performance of several predictors on CASP10. We show average value for precision, balanced accuracy (bacc), Mattehews correlation coefficient (MCC), and Area under the ROC curve (AUC).

Predictor	Precision	Bacc	MCC	AUC
Iupred (long)	0.231	0.575	0.145	0.621
Iupred (short)	0.413	0.729	0.374	0.712
SPINE-D	0.307	**0.779**	0.366	0.876
DisoPred3	**0.607**	0.719	0.467	0.883
DeepCNF-D	0.529	0.764	**0.474**	**0.898**
DeepCNF-D (ami_only)	0.504	0.737	0.433	0.772

We also compare our method with other predictors using precision, balanced accuracy (bacc), and Mattehews correlation coefficient (MCC), respectively. Since these measurements are based on a user-defined threshold for order/disorder classification, we use default value (*i.e.*, 0.5) for the other predictors while choosing 0.2 for our method DeepCNF-D. The reason to choose 0.2 for our method is based on the optimal performance of MCC value on the training data. The results show that DeepCNF-D archives 0.486 MCC value on CASP9 and 0.474 on CASP10, which are higher than the other predictors. If only amino acid related features are used, DeepCNF-D (ami_only) reaches 0.4 MCC on CASP9 and 0.433 on CASP10, which are significantly higher than IUpred.

## 3. Conclusions and Discussions

A sequence labeling machine learning model, namely weighted DeepCNF (Deep Convolutional Neural Fields), for protein order/disorder prediction has been presented. This model not only captures long-range sequence information by a deep hierarchical architecture and exploits interdependency between adjacent order/disorder labels, but also assigns different weights for each label during training and prediction to solve the label imbalance issue that was known as a long-standing problem in order/disorder prediction. The source code of our method DeepCNF-D is available at http://ttic.uchicago.edu/~wangsheng/DeepCNF_D_package_v1.00.tar.gz [30]. The overall performance in terms of AUC value of DeepCNF-D reaches 0.855 on CASP9 and 0.898 on CASP10, which are better than the state-of-the-art methods.

It should also be noted that if amino acid related features are used, our method outperforms the best sequence-based method IUpred, while still keeps the same extremely fast running speed. This pure sequence version of our method (called DeepCNF-D ami_only) can be applied to large-scale proteome analysis for disordered regions [31]. From a computational perspective, our weighted DeepCNF model provides a new framework to incorporate long-range sequence information to predict the labels. Such a framework can be directly applied to many sequence labeling issues such as secondary structure [21], solvent accessibility [22], and structural alphabet [32,33,34,35,36].

The observation of the deterioration with three layers compared to two layers may due to over-fitting because the regularization factor is the same for all trained models. If we specifically fine-tune the regularization factor for the three layer model, then the results could be comparable to the two layer model. We also tried different numbers of neurons per hidden layer (from 10 to 100) for the one to three layers models. The improvements in terms of AUC saturated at 50 neurons and did not largely increase after 50. However, instead of using these ad-hoc fine-tune procedures, we could try to use the following general strategies to prevent over-fitting and to further improve the performance of the deep network architecture: (a) a dropout framework [32,33,34,35,36] which takes into consideration the hidden structures of the neurons; (b) a dimension reduction technique [37] which could be used to reduce the total number of neuron weights; (c) a hessian-free strategy [38] which could be applied to accelerate the calculation based on the reduced neuron space.

Further developments for single *ab initio* order/disorder prediction methods are still possible in the following three approaches: (a) Since the area under the curve (AUC) is a proper measurement for the performance of order/disorder prediction and was applied in the CASP assessment [39], a method directly optimizing the AUC value would have a high chance to increase the overall performance; (b) Besides the structure features used in our method, such as predicted secondary structure and solvent accessibility, further information could be incorporated into order/disorder prediction. As suggested in Ucon [32,33,34,35,36], the predicted residue-residue contact information could contribute to the prediction accuracy; (c) It is shown that the characteristics of terminal and non-terminal disorder regions differ a lot from each other [15], and it is the same case for long and short disorder regions [40]. In the future, maybe these different disorder regions could be assigned different labels and trained by weighted DeepCNF, since our model can handle the label imbalance issue.

## 4. Method

In this section we describe the definition of order/disorder states, our proposed prediction methods, as well as the related features for prediction. The first part is the definition for order/disorder regions on a given protein. The second part shows the major contribution of our work, a machine learning model called weighted DeepCNF (Deep Convolutional Neural Fields), for order/disorder region prediction. DeepCNF is a Deep Learning extension of a probabilistic graphical model Conditional Markov Neural Fields (CNF), which can capture medium- or even long-range information in a protein chain by a deep hierarchical architecture, and also model interdependency between adjacent order/disorder labels. In order to solve the label imbalance issue, we assign a relatively larger weight to disorder label, which is sparsely represented in the dataset. Then more cost would be given to errors in these disorder labels during training process to unbias the DeepCNF model. In the third part we introduce the related protein features that could be categorized into classes of amino acid, evolution, and structural properties.

### 4.1. Order/Disorder Definition

We use the same definition of order/disorder states as Cheng *et al.* [5] and the CASP organization [23]. In particular, segments longer than three residues but lacking atomic coordinates in the crystal structure are labeled “disordered” whereas all other residues are labeled “ordered”. Note that besides order/disorder state, there also exist “N” for not available and “X” for atomic coordinates missing in the CASP dataset. We simply remove these “N” residues and mark “X” residues as disordered.

### 4.2. DeepCNF Model

#### 4.2.1. Model Architecture

**Figure 1:**
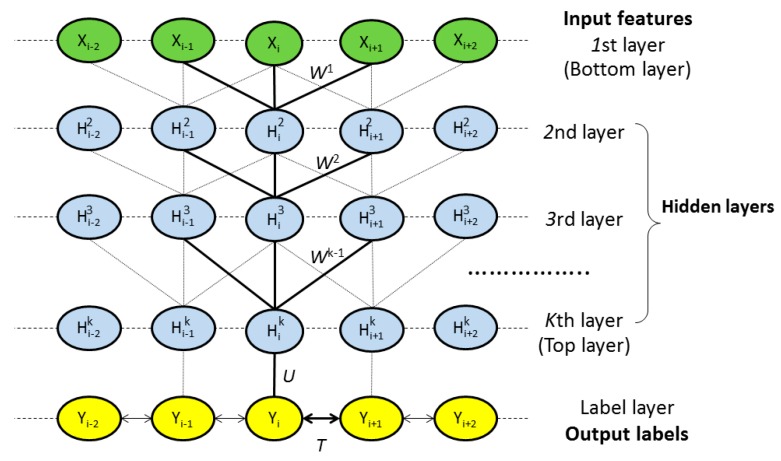
The architecture of DeepCNF, where i is the residue index, $X_{i}$ the associated input features, $H^{k}$ represents the *k*th hidden layer, and Y is the output label. All the layers from the 1st to the top layer form a deep convolutional neural network (DCNN). The top layer and the label layer form a conditional random field (CRF). $W^{k}\left\{k=1,2,\ldots ,K\right\}$, U and T are the model parameters where T is used to model correlation among adjacent residues.

As shown in Figure 1, DeepCNF consists of two modules: (a) the CRF module consisting of the top layer and the label layer; and (b) the deep convolutional neural network (DCNN) module covering the bottom layer to the top layer. When only one hidden layer is used, this DeepCNF becomes CNF, a probabilistic graphical model described by Peng *et al.* [15]. Given a protein sequence of length L, let $Y=\left(Y_{1},\ldots ,Y_{L}\right)$ denote its order/disorder label where $Y_{i}$ is the order/disorder state at residue i. Let $X=\left(X_{1},\ldots ,X_{L}\right)$ denote the input feature where $X_{i}$ is a column vector representing the input feature for residue i. Using DeepCNF, we calculate the conditional probability of Y on the input X as follows,

(1) $$P\left(Y\text{|}X\right)=\text{exp}\left(\overset{L}{\underset{i=1}{\sum }}\left[\Psi \left(Y,X,i\right)+\Phi \left(Y,X,i\right)\right]\right)/Z\left(X\right)$$

where Ψ(Y,X,i) is the potential function quantifying correlation among adjacent order/disorder states at around position i, Φ(Y,X,i) is the potential function modeling relationship between $Y_{i}$ and input features for position i , and Z(X) is the partition function. Formally, Ψ(Y,X,i) and Φ(Y,X,i) are defined as follows,

(2) $$\Psi \left(Y,X,i\right)=\underset{a,b}{\sum }T_{a,b}\delta \left(Y_{i}=a\right)\delta \left(Y_{i+1}=b\right)$$

(3) $$\Phi \left(Y,X,i\right)=\sum_{a}\sum_{j}U_{a,j}H_{j}\left(X,i,W\right)\delta \left(Y_{i}=a\right)$$

where a,b representing order/disorder states, say 0 for order and 1 for disorder. δ() is an indicator function, $H_{j}\left(X,i,W\right)$ is a neural network function for the j-th neuron at position i of the top layer, and W, U, and T are the model parameters to be trained. Specifically, W is the parameter for the neural network, U is the parameter connecting the top layer to the label layer, and T is for label correlation. The section below shows the details of the deep convolutional neural network corresponding to $H_{j}\left(X,i,W\right)$.

#### 4.2.2. Deep Convolutional Neural Network

**Figure 2:**
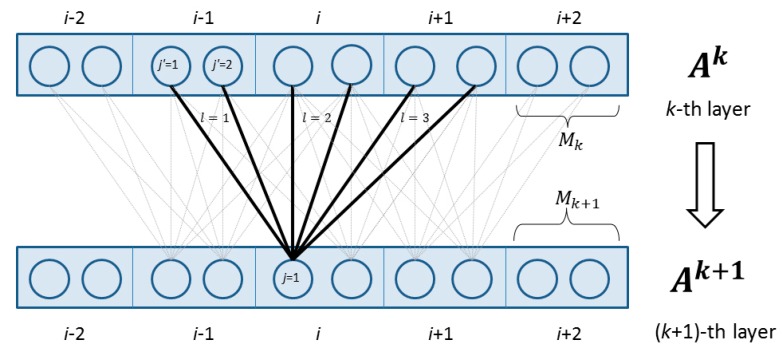
The feed-forward connection between two adjacent layers in the deep convolutional neural network.

Figure 2 shows two adjacent layers. Let $M_{k}$ be the number of neurons for a single position of the k-th layer. Let $X_{i}\left(j\right)$ be the j-th feature at the input layer for residue i and $A_{i}^{k}\left(j\right)$ denote the output value of the j-th neuron of position *i* at layer *k*. When k=1, $A^{k}$ is actually the input feature X. Otherwise, $A^{k}$ is a matrix of dimension $L\times \;M_{k}$. Let $2N_{k}+1$ be the window size at the k-th layer. Mathematically, $A_{i}^{k}\left(j\right)$ is defined as follows

(4) $$A_{i}^{k}\left(j\right)=X_{i}\left(j\right),\;\text{if}\;k=1$$

(5) $$A_{i}^{k+1}\left(j\right)=h\left(\sum_{l=-N_{k}}^{N_{k}}\sum_{j^{\prime }=1}^{M_{k}}\left(A_{i+l}^{k}\left(j^{\prime }\right)\text{*}W_{l}^{k}\left(j,j^{\prime }\right)\right)\right),\;\text{if}\;k<K$$

(6) $$H_{j}\left(X,i,W\right)=A_{i}^{K}\left(j\right)$$

Meanwhile, h is the activation function, either the sigmoid (*i.e.*, 1/(1+exp(−x))) or the tanh (*i.e.*, (1−exp(−2x))/(1+exp(−2x))) function. $W_{l}^{k}$ ($-N_{k}\leq l\leq N_{k}$) is a two-dimension weight matrix for the connections between the neurons of position i  at layer k and the neurons of position i+1 at layer k+1. $W_{l}^{k}$ is shared by all the positions in the same layer, so it is position-independent. Here j and j’ index two neurons at the k-th and (k+1)-th layers, respectively.

#### 4.2.3. Weighted DeepCNFs

Traditional algorithms choose an approximation of the accuracy (such as log probability of the occurrence label [19], or empirical label-wise accuracy [19]) as loss function. However, for imbalanced label applications, this criterion is no longer suitable since the minority label would have less impact on accuracy than the majority label, which could result in biased predictions [41]. Unfortunately, the order/disorder prediction is such a task whereas the minority class (*i.e.*, the disorder state) is of prime importance but this class might be completely ignored by traditional classifiers. For example, with an imbalance ratio of 0.93 to 0.07 for order/disorder states, a classifier that classifies everything to be ordered state will be 93% accurate, but it will be completely useless as a suitable classifier.

In order to solve this imbalanced label problem, one idea is to design cost-enabled classifiers that assign different weights for different labels [28]. In this way, more cost can be given to errors in the minority class to make the classifier unbiased. Formally, we redefine $\Psi^{\prime }\left(Y,X,i\right)$ and $\Phi^{\prime }\left(Y,X,i\right)$ functions to consider the weight of different labels as follows,

(7) $$\Psi^{\prime }\left(Y,X,i\right)=\underset{a,b}{\sum }T_{a,b}w_{a}\delta \left(Y_{i}=a\right)\delta \left(Y_{i+1}=b\right)$$

(8) $$\Phi^{\prime }\left(Y,X,i\right)=\sum_{a}\sum_{j}U_{a,j}H_{j}\left(X,i,W\right)w_{a}\delta \left(Y_{i}=a\right)$$

where $w_{a}$ is the weight of label a. In this work, we found out that the weight for order/disorder states could be set to the reciprocal of their occurrence frequency, *i.e*., 0.07 for order state and 0.93 for disorder state.

#### 4.2.4. Training Method, Regularization, and Time Complexity

We use the Limited-memory Broyden–Fletcher–Goldfarb–Shanno (L-BFGS) algorithm [28] as the optimization routine to search for the optimal model parameter that maximizes the object function (for more details, please refer to Supplemental Document). To control model complexity to avoid over-fitting, we add a L_2_-norm penalty term as the regularization factor. Suppose the number of neurons $M_{k}$ and the window size $N_{k}$ at each layer k are fixed for all layers as M and N, then the computational complexity of the gradient computation is $O\left(LKNM+LMD+LD^{2}\right)\;$ for a single input-output pair where the protein length is L and the label dimension D is 2.

### 4.3. Protein Features

A variety of protein features have been studied by Becker *et al.* [42] to predict the order/disorder states. These features could be categorized into three classes: amino acid related, evolution related, and structure related features, for each position i. Here we use 129 features, described below.

#### 4.3.1. Amino Acid Related Features

Four types of amino acid related features are considered, including: (a) amino acid indicator vector; (b) physico-chemical property; (c) specific propensity of being endpoints of a secondary structure segment; and (d) correlated contact potential. Specifically, the amino acid indicator vector is a 20 dimensional vector with value 1 for the specific amino acid occurring at position i while the other 19 values are 0; physico-chemical property has 7 values for each amino acid (shown in Table 1 from Meiler *et al.* [18]); specific propensity of being endpoints of an SS segment has 11 values for each amino acid (shown in Table 1 from Duan *et al.* [43]); correlated contact potential has 40 values for each amino acid (shown in Table 3 from Tan *et al.* [44]). All these features have been studied in Wang *et al.* [45] for secondary structure elements prediction, in Ma *et al.* [21] for solvent accessibility prediction, and in [22] for homology detection. Overall, at each position i, there are 78 = 20 + 7 + 11 + 40 amino acid related features.

#### 4.3.2. Evolution Related Features

The order/disorder state of a residue has a strong relationship with the residue’s substitution and evolution [46,47,48]. Residues in ordered state and disordered state were shown to have different substitution patterns due to different selection pressures. Evolutionary information such as PSSM (position specific scoring matrix) generated by PSI-BLAST [2] have been widely accepted to efficiently enhance prediction performance [49]. Here we use an additional evolution information from the HHM file generated by HHpred [18]. Overall, for each residue, there are 40 = 20 + 20 evolution related features.

#### 4.3.3. Structure Related Features

Local structural features, such as secondary structure and/or solvent accessibility, are also very useful for predicting order/disorder state, as indicated in [50]. Here we use the predicted eight-state secondary structure elements (SSE) [18] and three-state solvent accessibility (ACC) [21] probability as structure related features for each residue position. Overall, for each residue, there are 11 = 8 + 3 structure related features.

## Supplementary Materials

Supplementary materials can be found at http://www.mdpi.com/1422-0067/16/08/17315/s1.

## References

[1] Jirgensons B. (1958). Optical rotation and viscosity of native and denatured proteins. X. Further studies on optical rotatory dispersion. Arch. Biochem. Biophys..

[2] Oldfield C.J., Dunker A.K. (2014). Intrinsically disordered proteins and intrinsically disordered protein regions. Annu. Rev. Biochem..

[3] Dunker A.K., Oldfield C.J., Meng J., Romero P., Yang J.Y., Chen J.W., Vacic V., Obradovic Z., Uversky V.N. (2008). The unfoldomics decade: An update on intrinsically disordered proteins. BMC Genom..

[4] Jensen M.R., Ruigrok R.W., Blackledge M. (2013). Describing intrinsically disordered proteins at atomic resolution by NMR. Curr. Opin. Struct. Biol..

[5] He B., Wang K., Liu Y., Xue B., Uversky V.N., Dunker A.K. (2009). Predicting intrinsic disorder in proteins: An overview. Cell Res..

[6] Dosztányi Z., Csizmok V., Tompa P., Simon I. (2005). IUPred: Web server for the prediction of intrinsically unstructured regions of proteins based on estimated energy content. Bioinformatics.

[7] Jones D.T., Cozzetto D. (2015). DISOPRED3: Precise disordered region predictions with annotated protein-binding activity. Bioinformatics.

[8] Zhang T., Faraggi E., Xue B., Dunker A.K., Uversky V.N., Zhou Y. (2012). SPINE-D: Accurate prediction of short and long disordered regions by a single neural-network based method. J. Biomol. Struct. Dyn..

[9] Eickholt J., Cheng J. (2013). DNdisorder: Predicting protein disorder using boosting and deep networks. BMC Bioinform..

[10] Ishida T., Kinoshita K. (2007). PrDOS: Prediction of disordered protein regions from amino acid sequence. Nucleic Acids Res..

[11] Xue B., Dunbrack R.L., Williams R.W., Dunker A.K., Uversky V.N. (2010). PONDR-FIT: A meta-predictor of intrinsically disordered amino acids. Biochim. Biophys. Acta.

[12] Hirose S., Shimizu K., Kanai S., Kuroda Y., Noguchi T. (2007). POODLE-L: A two-level SVM prediction system for reliably predicting long disordered regions. Bioinformatics.

[13] Kozlowski L.P., Bujnicki J.M. (2012). MetaDisorder: A meta-server for the prediction of intrinsic disorder in proteins. BMC Bioinform..

[14] Deng X., Eickholt J., Cheng J. (2012). A comprehensive overview of computational protein disorder prediction methods. Mol. BioSyst..

[15] Monastyrskyy B., Kryshtafovych A., Moult J., Tramontano A., Fidelis K. (2014). Assessment of protein disorder region predictions in CASP10. Proteins Struct. Funct. Bioinform..

[16] Wang L., Sauer U.H. (2008). OnD-CRF: Predicting order and disorder in proteins conditional random fields. Bioinformatics.

[17] Lafferty J., McCallum A., Pereira F.C. Conditional random fields: Probabilistic models for segmenting and labeling sequence data. Proceedings of the 18th International Conference on Machine Learning (ICML-2001).

[18] Becker J., Maes F., Wehenkel L. (2013). On the encoding of proteins for disordered regions prediction. PLoS ONE.

[19] Peng J., Bo L., Xu J. Conditional neural fields. Proceedings of the Advances in Neural Information Processing Systems.

[20] Lee H., Grosse R., Ranganath R., Ng A.Y. Convolutional deep belief networks for scalable unsupervised learning of hierarchical representations. Proceedings of the 26th Annual International Conference on Machine Learning, 2009, ACM.

[21] Wang Z., Zhao F., Peng J., Xu J. (2011). Protein 8-class secondary structure prediction using conditional neural fields. Proteomics.

[22] Ma J., Wang S. (2015). AcconPred: Predicting solvent accessibility and contact number simultaneously by a multitask learning framework under the conditional neural fields model. BioMed Res. Int..

[23] Cheng J., Sweredoski M.J., Baldi P. (2005). Accurate prediction of protein disordered regions by mining protein structure data. Data Min. Knowl. Discov..

[24] Di Domenico T., Walsh I., Martin A.J., Tosatto S.C. (2012). MobiDB: A comprehensive database of intrinsic protein disorder annotations. Bioinformatics.

[25] Monastyrskyy B., Fidelis K., Moult J., Tramontano A., Kryshtafovych A. (2011). Evaluation of disorder predictions in CASP9. Proteins Struct. Funct. Bioinform..

[26] Faraggi E., Zhang T., Yang Y., Kurgan L., Zhou Y. (2012). SPINE X: Improving protein secondary structure prediction by multistep learning coupled with prediction of solvent accessible surface area and backbone torsion angles. J. Comput. Chem..

[27] Fawcett T. (2004). ROC graphs: Notes and practical considerations for researchers. Mach. Learn..

[28] De Lannoy G., François D., Delbeke J., Verleysen M. (2012). Weighted conditional random fields for supervised interpatient heartbeat classification. IEEE Trans. Biomed. Eng..

[29] Wang G., Dunbrack R.L. (2003). PISCES: A protein sequence culling server. Bioinformatics.

[30] The Source Code of Method DeepCNF-D. http://ttic.uchicago.edu/~wangsheng/DeepCNF_D_package_v1.00.tar.gz.

[31] Ward J.J., Sodhi J.S., McGuffin L.J., Buxton B.F., Jones D.T. (2004). Prediction and functional analysis of native disorder in proteins from the three kingdoms of life. J. Mol. Biol..

[32] Wang S., Zheng W.-M. (2008). CLePAPS: Fast pair alignment of protein structures based on conformational letters. J. Bioinform. Computat. Biol..

[33] Wang S., Zheng W.-M. (2009). Fast multiple alignment of protein structures using conformational letter blocks. Open Bioinform. J..

[34] Wang S., Peng J., Xu J. (2011). Alignment of distantly related protein structures: Algorithm, bound and implications to homology modeling. Bioinformatics.

[35] Wang S., Ma J., Peng J., Xu J. (2013). Protein structure alignment beyond spatial proximity. Sci. Rep..

[36] Ma J., Wang S. (2014). Algorithms, applications, and challenges of protein structure alignment. Adv. Protein Chem. Struct. Biol..

[37] Srivastava N., Hinton G., Krizhevsky A., Sutskever I., Salakhutdinov R. (2014). Dropout: A simple way to prevent neural networks from overfitting. J. Mach. Learn. Res..

[38] Neyshabur B., Panigrahy R. (2013). Sparse matrix factorization.

[39] Martens J. Deep learning via Hessian-free optimization. Proceedings of the 27th International Conference on Machine Learning (ICML-10).

[40] Schlessinger A., Punta M., Rost B. (2007). Natively unstructured regions in proteins identified from contact predictions. Bioinformatics.

[41] Gross S.S., Russakovsky O., Do C.B., Batzoglou S. Training conditional random fields for maximum labelwise accuracy. Proceedings of the Advances in Neural Information Processing Systems.

[42] Liu D.C., Nocedal J. (1989). On the limited memory BFGS method for large scale optimization. Math. Program..

[43] Meiler J., Müller M., Zeidler A., Schmäschke F. (2001). Generation and evaluation of dimension-reduced amino acid parameter representations by artificial neural networks. Mol. Model. Ann..

[44] Duan M., Huang M., Ma C., Li L., Zhou Y. (2008). Position-Specific residue preference features around the ends of helices and strands and a novel strategy for the prediction of secondary structures. Protein Sci..

[45] Tan Y.H., Huang H., Kihara D. (2006). Statistical potential-based amino acid similarity matrices for aligning distantly related protein sequences. Proteins Struct. Funct. Bioinform..

[46] Ma J., Wang S., Zhao F., Xu J. (2013). Protein threading using context-specific alignment potential. Bioinformatics.

[47] Ma J., Peng J., Wang S., Xu J. (2012). A conditional neural fields model for protein threading. Bioinformatics.

[48] Ma J., Wang S., Wang Z., Xu J. (2014). MRFalign: Protein homology detection through alignment of markov random fields. PLoS Comput. Biol..

[49] Altschul S.F., Madden T.L., Schäffer A.A., Zhang J., Zhang Z., Miller W., Lipman D.J. (1997). Gapped BLAST and PSI-BLAST: A new generation of protein database search programs. Nucleic Acids Res..

[50] Söding J., Biegert A., Lupas A.N. (2005). The HHpred interactive server for protein homology detection and structure prediction. Nucleic Acids Res..

