# Supplementary material for: DeepCNF-D: Predicting Protein Order/Disorder Regions by Weighted Deep Convolutional Neural Fields
Source: Int J Mol Sci. 2015 Jul 29;16(8):17315–30. doi: 10.3390/ijms160817315 (PMC4581195; doi:10.3390/ijms160817315)
Supplement: Supplementary file 1 [file ijms-16-17315-s001.zip › ijms-88480-Supplementary Information/DeepCNF_D_Supplemental_Material/ijms-88480-Supplementary Information.pdf]

Supplemental Information

1. DeepCNF Model

As shown in Supplemental Figure S1, DeepCNF consists of two modules: (a) the Conditional Random Fields (CRF) [17] module consisting of the top layer and the label layer; and (b) the deep convolutional neural network (DCNN) [20] module covering the input to the top layer. When only one hidden layer is used, this DeepCNF becomes Conditional Neural Fields (CNF), a probabilistic graphical model described in [19].

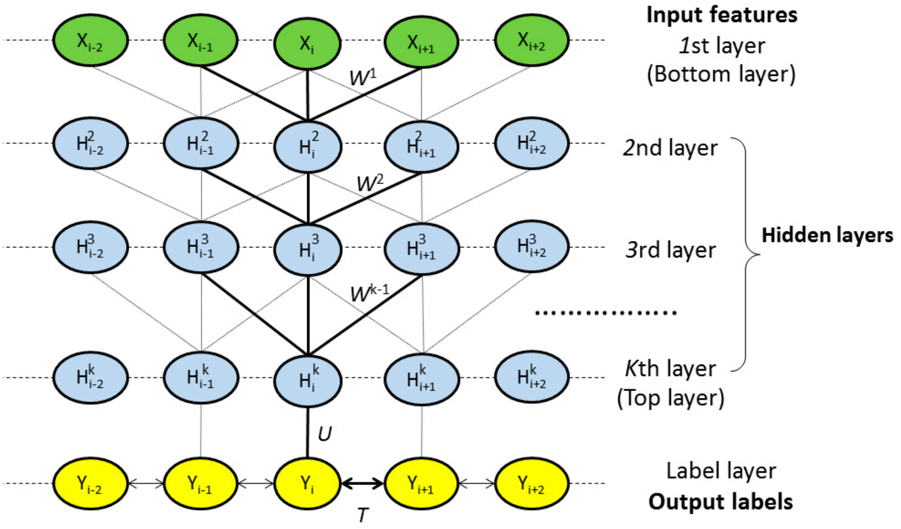

Figure S1. The architecture of DeepCNF, where i is the residue index, X_i the associated input features, H^k represents the kth hidden layer, and Y is the output label. All the layers from the 1st to the top layer form a deep convolutional neural network (DCNN). The top layer and the label layer form a conditional random field (CRF). $W^k\{k = 1,2, \dots, K\}$, U and T are the model parameters where T is used to model correlation among adjacent residues.

1.1. Conditional Random Field (CRF)

Given a protein sequence of length L, let $\boldsymbol{Y} = (Y_1, \dots, Y_L)$ denote its SS where Y_i is the SS type at residue i. Let $\boldsymbol{X} = (X_1, \dots, X_L)$ denote the input feature where X_i is a column vector representing the input feature for residue i. Using DeepCNF, we calculate the conditional probability of $\boldsymbol{Y}$ on the input $\boldsymbol{X}$ as follows,

$$P(\boldsymbol{Y}|\boldsymbol{X}) = \exp\left(\sum_{i=1}^{L}[\Psi(\boldsymbol{Y},\boldsymbol{X},i) + \Phi(\boldsymbol{Y},\boldsymbol{X},i)]\right)/Z(\boldsymbol{X}) \quad (1)$$

where $\Psi(\boldsymbol{Y},\boldsymbol{X},i)$ is the potential function quantifying correlation among adjacent SS types at around position i, $\Phi(\boldsymbol{Y},\boldsymbol{X},i)$ is the potential function modeling relationship between Y_i and input features for position i, and $Z(\mathbf{X})$ is the partition function. Formally, $\Psi()$ and $\Phi()$ are defined as follows,

$$\Psi(\boldsymbol{Y},\boldsymbol{X},i) = \sum_{a,b} T_{a,b}\delta(Y_i = a)\delta(Y_{i+1} = b) \quad (2)$$

$$\Phi(\boldsymbol{Y},\boldsymbol{X},i) = \sum_{a}\sum_{m} U_{a,m} H_m(\boldsymbol{X},i,W)\delta(Y_i = a) \tag{3}$$

where a and b represent secondary structure states, δ() is an indicator function, $H_m(\boldsymbol{X},i,W)$ is a neural network function for the m-th neuron at position i of the top layer, and W, U, and T are the model parameters to be trained. Specifically, W is the parameter for the neural network, U is the parameter connecting the top layer to the label layer, and T is for label correlation. Below see the details of the deep convolutional neural network for $H_m(\boldsymbol{X},i,W)$.

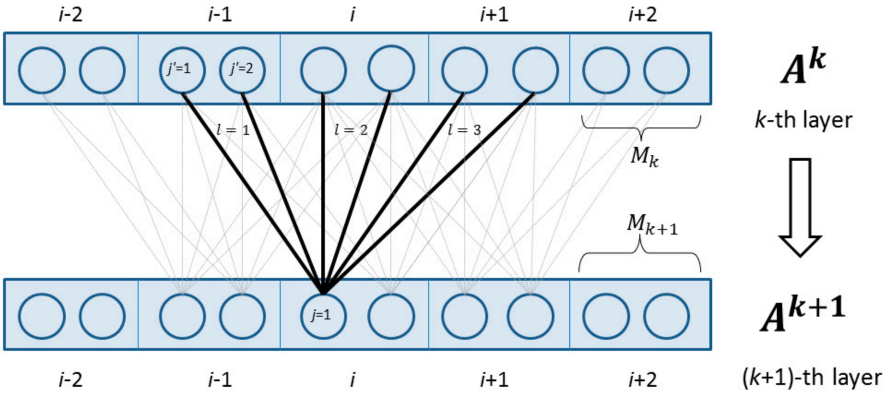

Figure S2. The feed-forward connection between two adjacent layers in the deep convolutional neural network.

1.2. Deep Convolutional Neural Network (DCNN)

Supplemental Figure S2 shows two adjacent layers. Let M_k be the number of neurons for a single position at the k-th layer. Let $X_i(m)$ be the m-th feature at the input layer for residue i and $A_i^k(m)$ denote the output value of the m-th neuron of position i at layer k. When $k = 1$, $\boldsymbol{A^k}$ is actually the input feature $\boldsymbol{X}$. Otherwise, $\boldsymbol{A^k}$ is a matrix of dimension $L \times M_k$. Let $2N_k + 1$ be the window size at the k-th layer. Mathematically, $A_i^k(m)$ is defined as follows.

$$\begin{aligned} &A_i^k(m) = X_i(m), && \text{if } k = 1; \\ &A_i^{k+1}(m) = h(\textstyle\sum_{n=-N_k}^{N_k}\sum_{m'=1}^{M_k}[A_{i+n}^k(m') * W_n^k(m,m')]), && \text{if } k < K; \\ &H_m(\boldsymbol{X},i,W) = A_i^k(m), && \text{if } k = K \end{aligned} \tag{4}$$

Meanwhile, $h()$ is the activation function, either the sigmoid (*i.e.*, $1/(1+\exp(-x))$) or the tanh (*i.e.*, $(1-\exp(-2x))/(1+\exp(-2x))$) function. W_n^k where $(-N_k \le n \le N_k)$ is a 2D weight matrix for the connections between the neurons of position $i+n$ at layer k and the neurons of position i at layer $k+1$. $W_n^k(m,m')$ is shared by all the positions in the same layer, so it is position-independent. Here m' and m index two neurons at the k-th and $(k+1)$-th layers, respectively.

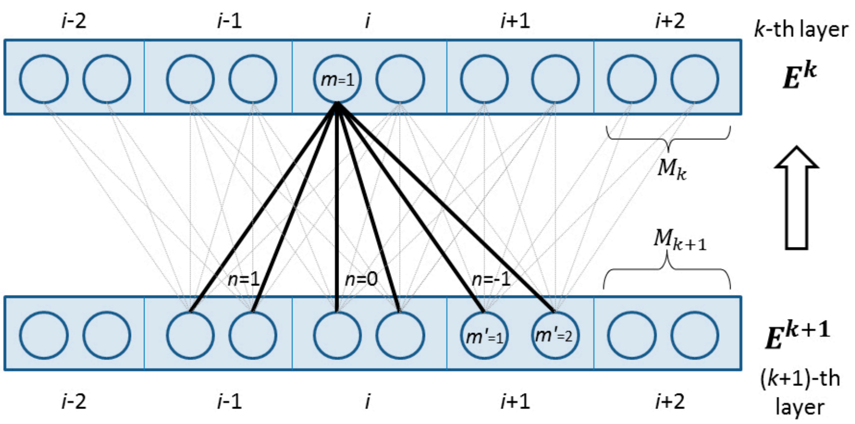

Figure S3. Illustration of calculating the gradient of deep convolutional neural network from layer $k+1$ to layer k.

2. Training Method

Similar to CRF [17], we train the model parameters by maximum-likelihood. The log-likelihood is as follows.

$$\log P(\boldsymbol{Y}|\boldsymbol{X}) = \sum_{i=1}^{L}[\Psi(\boldsymbol{Y},\boldsymbol{X},i) + \Phi(\boldsymbol{Y},\boldsymbol{X},i)] - \log Z(\boldsymbol{X}) \tag{5}$$

To train the model parameters, we need to calculate the gradient with respect to each parameter. We calculate the gradient first for CRF and then for DCNN. The gradient of the log-likelihood with respect to the parameters T and U is given by,

$$\begin{aligned}\nabla_{T_{a,b}} = &\left[\sum_{i=1}^{L}\delta(Y_i = a)\delta(Y_{i+1} = b)\right] \\ &- EXP_{P(\tilde{\boldsymbol{Y}}|X,W,U,T)}\left[\sum_{i=1}^{L}\delta(\tilde{Y}_i = a)\delta(\tilde{Y}_{i+1} = b)\right]\end{aligned} \tag{6}$$

$$\begin{aligned}\nabla_{U_{a,m}} = &\left[\sum_{i=1}^{L}\delta(Y_i = a)H_m(\boldsymbol{X},i,W)\right] \\ &- EXP_{P(\tilde{\boldsymbol{Y}}|\boldsymbol{X},W,U,T)}\left[\sum_{i=1}^{L}\delta(\tilde{Y}_i = a)H_m(\boldsymbol{X},i,W)\right]\end{aligned} \tag{7}$$

where EXP is the expectation function and can be calculated efficiently using the forward-backward algorithm [19]. As shown in Supplemental Figure S3, we can calculate the neuron error values at the k-th layer in a back-propagation mode as follows.

$$\begin{aligned}&E_i^k(m) = g(A_i^k(m)) * \textstyle\sum_a[E_i(a) * U_{a,m}], \qquad \text{if } k = K; \\ &\quad\text{where } E_i(a) = [\delta(Y_i = a)] - EXP_{P(\tilde{\boldsymbol{Y}}|\boldsymbol{X},W,U,T)}[\delta(\tilde{Y}_i = a)]; \\ &E_i^k(m) = g(A_i^k(m)) * \textstyle\sum_{n=-N_k}^{N_k}\sum_{m'=1}^{M_{k+1}}[E_{i-n}^{k+1}(m') * W_n^k(m',m)], \qquad \text{if } k < K\end{aligned} \tag{8}$$

where $g()$ is the derivative of the activation function; it is $g(x) = (1-x)x$ and $g(x) = 1 - x^2$ for the sigmoid and tanh function, respectively. $\boldsymbol{E^k}$ is the neuron error value matrix at the k-th layer, with dimension $L \times M_k$. $E_i(a)$ is the error of the log-likelihood function with respect to the label at the i-th

position and can be calculated by the forward-backward algorithm. Finally, the gradient of the parameter W at the k-th layer is:

$$\nabla_{W_n^k(m,m')} = \sum_{i=1}^{L} \left[E_i^{k+1}(m) * A_{i+n}^k(m')\right] \tag{9}$$

3. L_2 Regularization and L-BFGS

To reduce over-fitting, the log-likelihood objective function is penalized with a L_2-norm of the model parameters. Thus, our final objective function is as follows.

$$\max_\theta \log P_\theta(\boldsymbol{Y}|\boldsymbol{X}) - \lambda||\theta||^2 \tag{10}$$

where θ is the set of model parameters and λ is the regularization factor used to avoid overfitting. Although DeepCNF has a large number of model parameters, by setting the regularization factor large enough, we can make the L_2-norm of the model parameters small and thus, restrict the search space of the model parameter and avoid overfitting. However, a very large regularization factor (e.g., infinity) may restrict the model parameter into too small a search space and the resultant model may not learn enough from the training data (*i.e.*, under-fitting). We will determine the regularization factor by cross-validation.

Since the log-likelihood function is not convex, usually we can only solve the objective function to a local instead of global optimum. Although a typical way to train a deep network is to do it layer-by-layer, in our implementation we train all the model parameters simultaneously. We use the L-BFGS [42] to search for the optimal model parameters, which has also been successfully used to train CRF and CNF. In addition to learning the model parameters simultaneously, we can also train them layer-by-layer in a supervised mode. Starting from the first layer (*i.e.*, input feature), we train the model parameter $W1$ by removing the third to the K-th layers but keeping the label layer. After $W1$ is trained, we generate the neuron output values for the second layer and use them as input to train the model parameter $W2$ by removing the fourth to the K-th layers but keeping the label layer. We repeat this procedure until all the parameters are trained, and finally we fine-tune these parameters by simultaneous training.
